# The Incidence of and Risk Factors for Breast Infections After Tissue Expander Placement

**DOI:** 10.1093/asjof/ojad027.014

**Published:** 2023-04-14

**Authors:** Ameya Chumble, Thalia Anderson, Tindal McLaurin, Chase Schlesselman, Robin Kruse, Stephen Colbert

**Affiliations:** University of Missouri- Columbia, Columbia, MO; University of Missouri School of Medicine, Columbia, MO; University of Missouri- Columbia, Columbia, MO; University of Missouri- Columbia, Columbia, MO; University of Missouri- Columbia, Columbia, MO; University of Missouri- Columbia, Columbia, MO

## Abstract

**Goals/Purpose:**

Breast tissue expansion has a reported infection rate ranging from 2.4-25% in the literature when used for post mastectomy breast reconstruction. Expander infections may prolong the reconstructive timeframe, increase costs, portend poorer aesthetic outcomes, and decrease patient satisfaction. Medical treatments are often attempted to salvage implants prior to surgical treatment including irrigation, implant replacement, or explantation. The purposes of this study include 1) to ascertain the incidence of breast infections in patients with breast tissue expanders at our institution, 2) explore the success of medical treatments in precluding explantation, 3) identify modifiable risk factors associated with any post-expander breast infection, and 4) identify modifiable risk factors associated with post-expander breast infections requiring subsequent explantation.

**Methods/Technique:**

A single-center, single-surgeon, 4-year retrospective review was performed. Patient demographics, medical comorbidities, and surgical techniques were extracted. Clinical infection was defined by clinical evidence of cellulitis or abscess in a breast with a tissue expander present, regardless of the presence or absence of systemic symptoms. Preservation of the tissue expander following treatment of clinical infection with either antibiotics or surgical intervention was considered salvage, whereas removal of the expander with pocket closure was considered explantation. The incidence of infection and explantation were calculated separately. Chi-squared analysis was used to identify risk factors for both infection and explantation separately. A generalized estimated equations model was used to account for patients who had one infected breast and how that might affect the risk of infection in the other breast or in either breast following future implant or expander placement. It was also used to identify odds ratios for infection and explantation related to the risk factors identified previously.

**Results/Complications:**

A total of 349 breasts with tissue expanders in 163 patients were included in the study cohort. There was a 17.1% incidence of infection in the cohort and a 12.9% incidence of explantation secondary to infection. Of the infected breasts, 24.6% were managed with antibiotics alone. Significant risk factors for infection were prior chest radiation (p=0.021; OR 3.73, CI95% 1.22-11.34) and use of immune modulating drugs (p=0.012; 2.81, CI95% 1.25-6.30). Significant risk factors for explantation were age (p=0.030), tissue expander volume (p=0.038), and subpectoral placement (p=0.012). For each 5-year increase in age, odds ratio for explantation was 1.44 (CI95% 1.04-2.01). For every 50-cc increase in tissue expander volume, odds ratio for explantation was 1.28 (CI95% 1.02–1.55). Subpectoral placement carried an odds ratio of 11.5 (CI95% 1.7-77.3) compared to prepectoral placement or placement of a tissue expander under autologous tissue transfer (latissimus or free tissue transfer).

**Conclusion:**

The incidence of breast tissue expander related infections at our institution was within the ranges previously published. A quarter of the breasts with infections were able to successfully avoid explantation with prompt initiation of antibiotic therapy. A history of chest radiation prior to mastectomy and the use of immune modulating drugs were noted to be significant risk factors for developing infections in breasts with tissue expanders. Explantation was more common with increases in age and tissue expander volumes. Surprisingly, subpectoral placement portended a significantly higher risk of explantation as compared to prepectoral tissue expander placement or when placed under a flap. BMI, a history of smoking, intent of mastectomy (prophylactic vs curative), adjuvant radiation, and use of acellular dermal matrix were not significantly related to either infection or explantation in this cohort.

